# Comparison of Wearable and Depth-Sensing Technologies with Electronic Walkway for Comprehensive Gait Analysis

**DOI:** 10.3390/s25175501

**Published:** 2025-09-04

**Authors:** Marjan Nassajpour, Mahmoud Seifallahi, Amie Rosenfeld, Magdalena I. Tolea, James E. Galvin, Behnaz Ghoraani

**Affiliations:** 1 Department of Computer and Electrical Engineering and Computer Science, Florida Atlantic University, Boca Raton, FL 33431, USA; mnassajpoure2022@fau.edu (M.N.); mseifallahi2022@fau.edu (M.S.); 2 Comprehensive Center for Brain Health, Department of Neurology, University of Miami, Boca Raton, FL 33433, USAjeg200@med.miami.edu (J.E.G.)

**Keywords:** gait analysis, wearable sensors, Azure Kinect depth camera, Zeno™ walkway, inertial measurement units (IMUs), digital biomarkers, functional biomarkers

## Abstract

Accurate and scalable gait assessment is essential for clinical and research applications, including fall risk evaluation, rehabilitation monitoring, and early detection of neurodegenerative diseases. While electronic walkways remain the clinical gold standard, their high cost and limited portability restrict widespread use. Wearable inertial measurement units (IMUs) and markerless depth cameras have emerged as promising alternatives; however, prior studies have typically assessed these systems under tightly controlled conditions, with single participants in view, limited marker sets, and without direct cross-technology comparisons. This study addresses these gaps by simultaneously evaluating three sensing technologies—APDM wearable IMUs (tested in two separate configurations: foot-mounted and lumbar-mounted) and the Azure Kinect depth camera—against ProtoKinetics Zeno™ Walkway Gait Analysis System in a realistic clinical environment where multiple individuals were present in the camera’s field of view. Gait data from 20 older adults (mean age 70.06±9.45 years) performing Single-Task and Dual-Task walking trials were synchronously captured using custom hardware for precise temporal alignment. Eleven gait markers spanning macro, micro-temporal, micro-spatial, and spatiotemporal domains were compared using mean absolute error (MAE), Pearson correlation (*r*), and Bland–Altman analysis. Foot-mounted IMUs demonstrated the highest accuracy (MAE =0.00–6.12, r=0.92–1.00), followed closely by the Azure Kinect (MAE =0.01–6.07, r=0.68–0.98). Lumbar-mounted IMUs showed consistently lower agreement with the reference system. These findings provide the first comprehensive comparison of wearable and depth-sensing technologies with a clinical gold standard under real-world conditions and across an extensive set of gait markers. The results establish a foundation for deploying scalable, low-cost gait assessment systems in diverse healthcare contexts, supporting early detection, mobility monitoring, and rehabilitation outcomes across multiple patient populations.

## 1. Introduction

Gait is fundamental indicators of overall health and functional mobility, and their quantitative assessment plays an increasingly critical role across a wide range of clinical applications. These include fall risk prediction, post-stroke rehabilitation, musculoskeletal disorders, and early detection of neurological diseases such as Alzheimer’s and Parkinson’s disease [1,2,3]. Traditionally, gait evaluations rely heavily on clinician observation or standardized tests conducted in controlled settings. However, studies have shown that gait parameters may vary significantly between structured clinical assessments and real-world environments, including outdoor or crowded spaces, underscoring the importance of conducting gait analysis in more naturalistic contexts [4,5]. While informative, these methods are inherently subjective, time-consuming, and dependent on expert availability—posing barriers to widespread, routine use, particularly in resource-limited or remote environments [6,7].

To overcome these limitations, the field has witnessed a surge in the development of sensing technologies that offer objective, precise, and scalable gait assessments. These systems range from optoelectronic motion capture tools (e.g., Vicon), to pressure-sensitive walkways (e.g., GAITRite or Zeno™ Walkways), to wearable inertial measurement units (IMUs), to markerless depth cameras (e.g., Kinect v2, Azure Kinect) [8,9,10]. Such technologies are increasingly used not only in research settings but also in clinical environments, enabling fine-grained analysis of spatiotemporal gait features that can support diagnosis, monitor disease progression, and evaluate therapeutic outcomes across diverse patient populations [11,12,13,14].

A growing body of literature has explored the validity and reliability of these technologies by comparing them to reference systems. For example, several studies have benchmarked wearable sensors against pressure-sensitive walkways [15,16,17,18,19,20,21], or compared depth cameras with motion capture systems and clinical standards to evaluate gait parameters in controlled environments [22,23,24,25,26,27]. However, these studies lack precise temporal synchronization across sensing modalities, compromising the accuracy of multimodal comparisons. Moreover, prior studies focus on a narrow set of gait metrics—typically averaging only a handful of spatiotemporal parameters such as stride length, step time, or velocity. While informative, such limited analyses may overlook more subtle but clinically relevant aspects of gait, particularly in applications requiring early or differential diagnosis—such as mild cognitive impairment, atypical Parkinsonian syndromes, or neuropathies like those associated with diabetes [28,29,30,31]. Furthermore, these studies often share critical limitations. Most collect data under highly controlled laboratory conditions with only the subject in view of the camera—conditions that fail to reflect the complexities of real-world clinical environments where caregivers, clinical staff, or assistive devices are often present [22,23,24,25,32]. This discrepancy limits the generalizability and clinical readiness of such systems. Despite these shortcomings, recent advances in sensor technologies—including soft, flexible, and high-resolution wearable devices—are helping to overcome prior limitations in cost, scalability, and long-term reliability of gait monitoring systems [33,34,35]. These innovations promise broader clinical and home-based applications by improving accuracy and usability. While our study focuses on widely adopted tools such as the Azure Kinect and foot/lumbar IMUs, future integration of next-generation sensors may further enhance precision and deployment flexibility.

In response to these limitations, this study makes several novel contributions aimed at advancing the clinical utility of sensor-based gait analysis systems. First, we developed and utilized a custom hardware-based system for automated, millisecond-level synchronization across three sensing platforms: the Zeno™ Walkway pressure-sensitive walkway (serving as the gold standard), APDM wearable IMUs, and the Azure Kinect depth camera. This synchronization ensures accurate multimodal alignment for reliable validation. Second, we expanded the analytic scope by extracting a rich set of gait markers—including two macro-level and nine micro-level parameters—providing a detailed profile of gait dynamics. This high-resolution marker set enables a more nuanced assessment of gait alterations associated with neurodegenerative disease risk and supports future work on digital biomarker discovery. Third, we conducted gait recordings in real-world clinical environments where multiple individuals—such as caregivers or staff—may be present within the depth camera’s field of view. This design introduces realistic visual complexity and tests the robustness of tracking algorithms under practical conditions. Collectively, this work advances the state of the art by demonstrating the feasibility, accuracy, and clinical relevance of depth cameras and wearable sensors for detailed gait analysis in realistic healthcare settings. The resulting multimodal, synchronized dataset and comprehensive marker set lay the groundwork for scalable, objective, and versatile tools capable of supporting diverse clinical needs, from diagnostics to long-term monitoring and beyond.

## 2. Materials and Methods

### 2.1. Participants and Recruitment

This study initially recruited 20 participants (12 females, 8 males), aged between 52 and 82 years. Participants were either enrolled through the Healthy Brain Initiative (HBI) [36] at the University of Miami’s Comprehensive Center for Brain Health (CCBH) or were members of the general public who expressed interest in the study. The inclusion criteria included the following: (1) age 21 years or older; (2) ability to walk independently, with or without a walking aid (e.g., cane, walker); (3) capacity to provide informed consent; (4) willingness to be video recorded during walking and movement assessments. The exclusion criteria were the following: (1) severe mobility impairments preventing safe ambulation even with assistive devices;(2) unstable medical conditions that could pose safety risks during participation (e.g., uncontrolled hypertension, recent surgery, or acute illness).

Each participant underwent a physical evaluation—including InBody body composition analysis and anthropometric measurements—as well as a cognitive assessment using the Montreal Cognitive Assessment (MoCA). Data from two participants were excluded due to technical issues, specifically poor signal quality caused by sensor displacement during walking trials. As a result, data from 18 participants were deemed suitable for further analysis. Table 1 summarizes the demographic characteristics of the participants whose data were successfully recorded and included in the final analysis. All procedures adhered to the principles of the Declaration of Helsinki and were approved by the Institutional Review Board (IRB) at Florida Atlantic University and University of Miami. Written informed consent was obtained from all participants prior to data collection after providing enough information about the study.

### 2.2. Study Design

**Figure 1:**
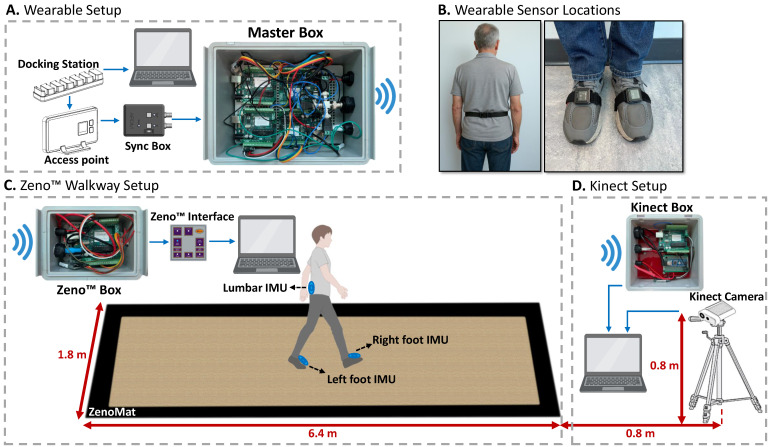
Designed wireless synchronization setup for APDM, Zeno™ Walkway, and Kinect systems. (**A**) The APDM system acts as the master, generating trigger signals via a Sync Box. (**B**) Sensor placement on lumbar (L5) and feet. (**C**) Signals are wirelessly sent to the Zeno™ Box to synchronize with the Zeno Interface. (**D**) Simultaneously, signals reach the Kinect Box to align camera recordings with the APDM timeline.

The participants wore three APDM IMUs, secured with straps and placed on the dorsal surface of the left and right feet, as well as the lower back at the level of the fifth lumbar vertebra (see Figure 1B). Each recording session included three tasks: a brief practice trial, followed by *Single-Task* and *Dual-Task* conditions. The practice trial, which followed the same walking pattern as the Single and Dual-Task, was conducted to familiarize participants with the procedure. In the Single-Task condition, participants performed straight, back-and-forth walking over approximately seven meters on the Zeno™ walkway, which is a pressure sensor integrated mat. In the Dual-Task condition, participants repeated the same walking route while performing a cognitive task—counting backward from 80 in steps of seven—a widely used paradigm in neurodegenerative research to impose additional cognitive load [37]. Dual-tasking typically results in slower walking speeds and altered gait dynamics, allowing evaluation of sensor performance across two distinct gait patterns within the same individuals. Two blue lines were marked on the outside of the Zeno™ Walkway, each placed one meter from the respective ends. The line at the starting end indicated both the start and end points, while the line at the opposite end marked the turning point. Participants began at the start line, walked to the turning line, turned around, and returned to the start line. The walking surface consisted of the Zeno™ Walkway, which features a flat, non-slip, industrial-grade vinyl surface integrated with pressure sensors. Before task execution, the Azure Kinect system was activated to record continuously throughout all tasks. The recording setups are described in detail in Section 2.3, Section 2.4, Section 2.5.

### 2.3. Recording Tools

Participants performed walking tasks while their gait was simultaneously recorded using three synchronized systems: a pressure-sensitive gait analysis mat, a wearable sensor system with IMUs, and a depth camera–based system. Among these, the Zeno™ Walkway—previously validated for accurate spatiotemporal gait assessment in similar applications [38]—served as the gold standard for evaluating and comparing gait measures derived from the other two systems.

#### 2.3.1. Pressure-Sensitive Gait Mat

Zen Electronic Walkway (Zeno™ Walkway; ProtoKinetics Inc., Havertown, PA, USA) contains a matrix of pressure-activated sensors. The system used in this study was 1.8 m in width and 6.4 m in length (activated width = 1.2 m, activated length = 6 m) and had a spatial and temporal resolution of 1.27 cm and 120 Hz, respectively. The raw pressure data were analyzed using PKMAS (Protokinetics Movement Analysis Software; ProtoKinetics Inc.), which automatically identifies gait events—such as heel strike (HS) and toe-off (TO)—and computes spatiotemporal gait parameters such as step length, stride length, cadence, and gait speed [38]. To ensure accurate comparisons, only gait cycles fully captured within the Zeno™ Walkway’s active sensing area were included in the analysis. The PKMAS software automatically excluded partial or off-mat steps, helping to eliminate boundary effects and ensure high-fidelity spatiotemporal data. This approach, consistent with prior validation studies [16,17,19,20], supports the use of Zeno™ Walkway as a reliable reference for gait analysis.

#### 2.3.2. Wearable Sensors Systems

Three inertial measurement units (APDM Opal, Portland, OR, USA) were used to capture motion data at a sampling frequency of 128 Hz. Sensors were positioned on both feet and the lumbar region. Two sensor configurations were evaluated: (1) a dual-sensor setup with foot-mounted sensors, and (2) a single-sensor setup with only the lumbar-mounted unit. To ensure consistent analysis and comparison across wearable sensors, a unified coordinate system was established. The Y-axis was oriented outward from the skin, the X-axis was defined by the right-hand rule, and the Z-axis, aligned with the sensor’s side buttons, pointed toward the ground, as detailed in [11]. Data acquisition was managed through APDM’s Motion Studio software, which facilitated sensor calibration, configuration, and real-time signal visualization. However, no proprietary APDM algorithms or software packages were used for gait parameter extraction. Instead, the raw IMU data were exported and analyzed offline using our custom algorithms, specifically designed to extract spatiotemporal gait features relevant to this study.

#### 2.3.3. Depth Camera

The Azure Kinect, Microsoft’s latest depth-sensing camera, was used to capture gait during the walking trials. Introduced in 2020 as the successor to Kinect v2, Azure Kinect utilizes time-of-flight technology to estimate pixel depth and offers improved spatial resolution and enhanced body tracking capabilities [39]. The device outputs three data streams—RGB, depth, and skeletal data—and can track up to 32 body joints [39]. The Azure Kinect was set to record the data up to 30 frames per second.

### 2.4. Synchronization Setup

To ensure precise temporal alignment across all recording systems—Zeno™ Walkway, APDM wearable sensors, and Azure Kinect—a custom hardware-based synchronization solution was developed. This system relied on a Bluetooth-enabled protocol to wirelessly transmit trigger signals to each device, coordinating data acquisition across the platforms. In this setup, the APDM system functioned as the master controller, as it supports the integration of a dedicated Sync Box into its wearable sensor configuration (Figure 1A). When the APDM system initiates data recording, the Sync Box outputs a 5-volt, TTL-level trigger signal via its output port. This signal serves as the master synchronization pulse and is transmitted wirelessly to the receiving systems—Zeno™ Walkway and Azure Kinect—using a custom-built Master Box.

The Zeno™ Walkway system receives the synchronization signal through a dedicated Zeno™ Box, which routes the signal to the SYNC INPUT port of the Zeno Interface (Figure 1C), thereby aligning its internal recording timeline with the APDM system. In parallel, the Kinect system receives the same signal via the Kinect Box (Figure 1D), which relays it to the Kinect’s computing interface, enabling synchronization of the depth camera data with the wearable sensor recordings. The APDM system offers the flexibility to output both edge-triggered and TTL-level signals. Since the Zeno™ Walkway’s Zeno Interface requires TTL-level signals and interprets signal states such that a low signal (0 V) denotes active recording and a high signal (5 V) indicates non-recording periods, the APDM Sync Box was configured accordingly. Specifically, it was set to emit a constant high-level signal during idle periods and to drop to a low-level signal during active task performance, ensuring compatibility with Zeno™ Walkway’s synchronization requirements.

### 2.5. Setup and Procedure

**Figure 2:**
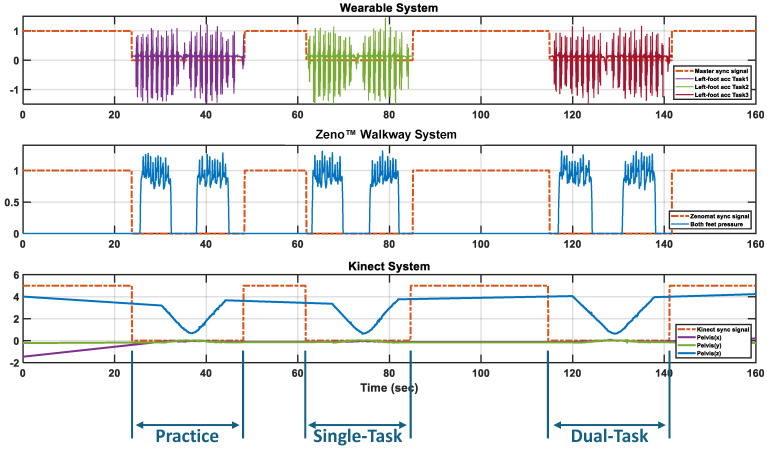
Synchronized trigger signals across APDM, Zeno™ Walkway, and Kinect systems during multiple tasks. The sync signal remains high when no task is active and drops low during task execution. APDM records only during active task periods, while Zeno™ Walkway and Kinect record continuously, using the sync signal as a temporal reference to identify the practice trial, Single-Task, and Dual-Task.

Figure 1 illustrates the complete setup used to collect synchronized gait data from wearable IMUs, the Zeno™ Walkway, and the Azure Kinect depth camera. Prior to task execution, the Zeno™ Walkway and Kinect systems were activated to record continuously throughout the practice, Single-Task, and Dual-Task walking conditions. Synchronization was controlled by sending trigger signals from the wearable sensor setup to the Zeno™ Walkway and Kinect at the beginning and end of each task. Figure 2 presents an example of the raw gait signals and synchronized trigger signals across all three systems for a single subject, demonstrating how the signal transitions from a high level during non-task periods to a low level during active walking. For the Zeno™ Walkway and Kinect systems, which recorded continuously, these triggers served as temporal markers to segment task-specific data, whereas the APDM system inherently aligned recordings with the active walking periods defined by the master synchronization signal.

### 2.6. Gait Marker Extraction

**Figure 3:**
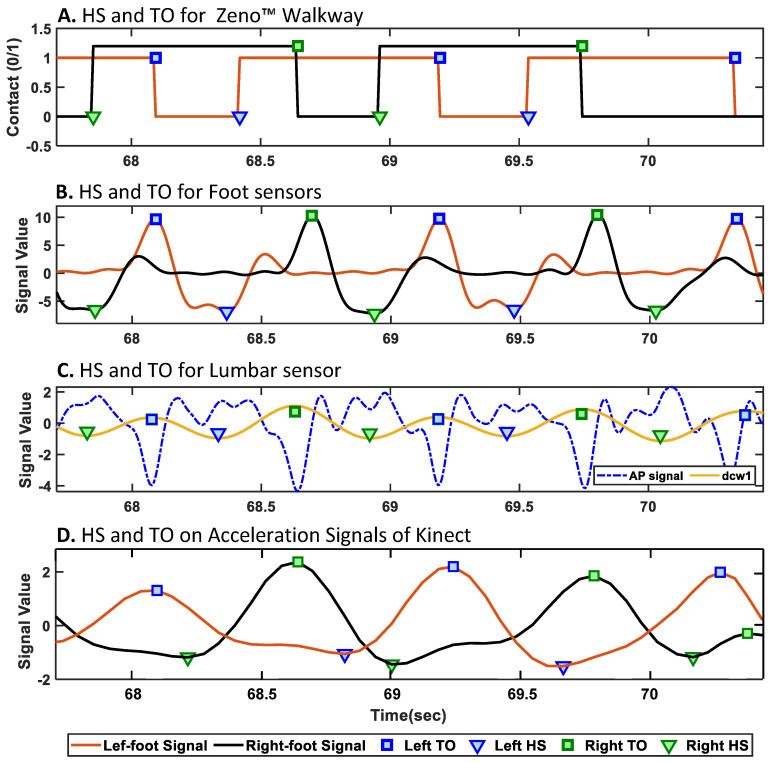
Detected gait events (heel strike [HS] and toe-off [TO]) for a representative subject across multiple modalities. (**A**) Zeno™ Walkway; (**B**) foot-mounted IMUs; (**C**) lumbar-mounted IMU; (**D**) acceleration signals extracted from heel joints by the Azure Kinect camera. Each subplot shows the automatically extracted HS and TO events using sensor-specific algorithms.

In this section, we describe the methodology used to extract temporal and spatial gait markers from the three technologies. The first step is to detect gait events—HS (Heel-Strike) and TO (Toe-Off)—from the recorded data of each system. For the Zeno™ Walkway system, the gait events and markers were derived using PKMAS software. Figure 3A illustrates the HS and TO events on the foot contact data for a representative subject using Zeno™ Walkway. It is important to note that all the gait events were independently extracted from wearable IMUs (lumbar and bilateral foot-mounted) and Kinect video recordings using custom algorithms tailored, as explained in Section 2.6.1, Section 2.6.2, Section 2.6.3. Section 2.6.4 then explains the calculation of temporal and spatial gait markers based on the detected events.

#### 2.6.1. HS/TO Detection with APDM Foot IMUs

HS and TO events were detected using the medial–lateral angular velocity (Y-axis gyroscope signal) from the foot-mounted inertial sensors [40]. The raw signal was first processed using a two-stage filtering pipeline. A 12th-order, high-pass Butterworth filter, implemented in second-order sections, was applied to remove low-frequency drift. This was followed by a custom FIR low-pass filter with 216 coefficients to attenuate high-frequency noise. The filtered signal was then mean-centered to obtain a clean oscillatory waveform suitable for event detection. HS events were identified as the negative peaks (minima), and TO events as the positive peaks (maxima) of the processed signal. Events were detected using MATLAB’s *findpeaks* function, with parameters set for minimum peak height, prominence, and inter-peak distance to ensure reliable identification. All analyses were conducted using MATLAB R2024a. This approach is entirely signal-based and does not involve strapdown integration. Figure 3B illustrates the detected HS and TO events for the left and right foot sensors for the representative subject.

#### 2.6.2. HS/TO Detection with APDM Lumbar IMU

The gait event detection approach was based on the method described in [41], with a modification to improve TO detection. The anterior–posterior acceleration signal (Z-axis of the accelerometer) was first preprocessed by linear detrending and low-pass filtering at 10 Hz using a second-order Butterworth filter. The filtered signal was then integrated using the cumtrapz function and subsequently differentiated using the continuous wavelet transform (CWT) with the cwt function in MATLAB. A Gaussian first-order (gaus1) wavelet was applied at an estimated optimal scale. Local minima and maxima of the first-order differentiated signal were identified as HS and TO events, respectively, using the findpeaks function. All analyses for the lumbar sensor were performed in MATLAB R2014a. Spatiotemporal gait parameters were then calculated from the timing of HS and TO events, along with step length estimations based on an inverted pendulum model, as described in [42]. Figure 3D shows the detected HS and TO events for the left and right foot estimated using the lumbar-mounted sensor.

#### 2.6.3. HS/TO Detection with Azure Kinect

**Figure 4:**
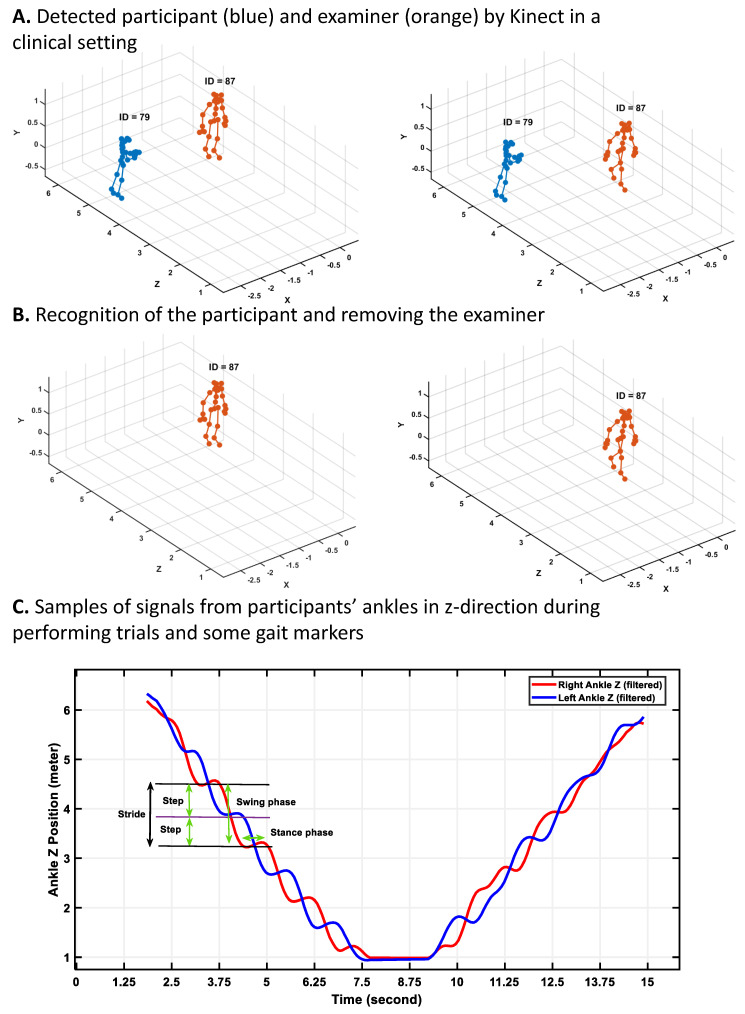
Processing Azure Kinect data in a real clinical setting with attention to other people for gait marker extraction. (**A**) Detected participant (blue) and examiner (orange) by Kinect in a clinical setting; (**B**) Recognition of the participant and removing the examiner; (**C**) Samples of signals from participants’ ankles in z-direction (depth) during performing trials and some gait markers. When the participant walks toward the camera, the z-values of the ankles decrease, and after turning and coming back toward the start point, the z-values increase.

In this study, the data were recorded via Azure Kinect in a real setting of the clinics, where there were other people in the recording environment. Thus, more than one person was in the angle of view of the Azure Kinect, and those people were also detected by the Azure Kinect (see Figure 4A). Also, as the data were recorded continuously in a synchronized way with other technologies, different IDs were assigned to the people who came and went out of the camera’s angle. Consequently, before working on the signals of the skeletal data of the participant to extract gait markers, several successive preprocessing steps were performed on the recorded data in the clinics.

First, using the annotation signals, various types of recorded tests were separated. Then, for each of the recorded tasks, the recorded skeletal data of people in the clinics tracked by Kinect were visualized. Our analysis showed that for the participant, the ID assigned by Azure Kinect did not change during each of the separate trials as the camera was set to track the participant’s body without missing it during the closing process (see Figure 4A). However, the assigned ID to other people who came into view and went out of the camera’s view changed. Regarding these findings and the location of the participants during the trials, we could separate the participants’ data from other people detected by Kinect. Figure 4 shows the samples of the skeletal data visualized via the MATLAB 2024a version, the recognition of the participants, and finally the plotting of signals of the right and left ankle of the participants during walking to detect gait cycles, strides, steps, subphases of gait cycles, etc.

After separating the skeletal data of the participants from other people tracked by Kinect, in the preprocessing stage (see Figure 4B), a sixth-order Butterworth filter with a 3 Hz cutoff frequency was applied to the joint movement signals to eliminate noise [43,44]. To detect the gait cycles and their related gait markers, such as stance and swing phases, step and stride times, and length, we need to detect the heel strike and toe-off occurrence, like other technologies (e.g., electronic walkways or IMU sensors). We applied the second gradient on the signals of the right and left ankles in the y-direction to convert the location signals of the ankle and foot to acceleration signals. We found the local maximum and minimum of the ankle acceleration signals as heel strikes and toe-off, respectively (see Figure 3D). Next, various gait markers were calculated using heel strike and toe-off incidents as described in the following section.

#### 2.6.4. Calculation of Gait Markers

Temporal and spatial gait parameters were calculated following TO and HS detection. The temporal gait markers (e.g., step time, stride time, stance time) were computed based on the timing of HS and TO events and are consistent across all sensing technologies. These formulas are presented in Table 2 for the right foot, assuming the left foot initiates the gait cycle; analogous formulas were applied to the left foot by adjusting the event sequence accordingly. In contrast, the spatial gait markers (e.g., step length, stride length) were derived using technology-specific methods that do not rely directly on HS and TO timing, and are therefore shown separately for each sensing technology in Table 2. The index *i* in the formulas shows the current events (i.e., current HS or TO), and i+1 shows the next event.

Step length from the foot-mounted sensors was estimated using an empirical equation, as proposed by Weinberg [45]. The calculation requires determining the vertical acceleration component ($a_{Z}$), derived from the foot’s local acceleration signals $a_{x}$ and $a_{z}$ using the relation $a_{Z}=a_{x}\sin\theta +a_{z}\cos\theta -g$, where θ is the pitch angle estimated from gyroscope data, and *g* is the gravitational acceleration [46]. The parameters $a_{\max}$ and $a_{\min}$ represent the maximum and minimum values of $a_{Z}$ within a single step, and *K* is a calibration constant used for subject-specific adjustment. Stride length was calculated using the same formula, but $a_{\max}$ and $a_{\min}$ were extracted over the duration of a full stride instead of a single step.

Step length from the lumbar-mounted sensor was estimated using the inverted pendulum model, as described in [42]. In this model, *h* denotes the vertical displacement of the center of mass, obtained through double integration of the vertical acceleration ($a_{x}$) using the cumulative trapezoidal method (cumtrapz). The parameter *l* represents the pendulum length, which was approximated by the subject’s foot length.

Step length from the Kinect is calculated by subtracting the z-direction position of one foot at its heel strike from the z-direction position of the opposite foot at the time of its toe-off. Also, to calculate the stride length, the location of the same foot during two successive heel strikes is subtracted from each other (see Table 2 for step and stride length formulas used to extract via Azure Kinect data).

### 2.7. Evaluation and Statistical Analysis

All statistical analyses were performed using MATLAB R2024a. Agreement between gait markers derived from Zeno™ Walkway and those obtained from APDM foot-mounted sensors, APDM lumbar-mounted sensors, and Azure Kinect was evaluated using Pearson correlation coefficient (*r*), mean absolute error (MAE) [11], and Bland–Altman analysis. *r* assessed the linear association between the systems, while MAE quantified the average magnitude of error. Bland–Altman plots were used to visualize the mean differences and 95% limits of agreement (LoA) between measurement methods, providing insight into systematic bias and variability. Statistical significance was defined as p<0.05 [47].

## 3. Results

The analysis was conducted across a range of gait markers categorized into macro- and micro-level parameters, where macro-level gait markers capture overall performance and include average gait velocity and cadence, and micro-level gait markers reflect more detailed aspects of the gait cycle. The micro-level markers were further classified into the following: Temporal markers (e.g., stride time, step time, stance time, swing time, single and double support time), Spatial markers (e.g., stride length, step length), and Spatiotemporal markers (e.g., stride velocity). These categories allowed for a comprehensive evaluation of each sensing technology’s ability to measure both high-level and fine-grained gait characteristics under both single-task and dual-task walking conditions.

Table 3 presents the results of the comparison for 11 extracted gait markers obtained using three different sensor technologies: the Zeno™ Walkway, APDM IMUs mounted on the feet and the lumbar region, and the Azure Kinect depth camera. For each gait marker, the mean ± standard deviation (Mean±SD) is reported along with the MAE, which was computed by comparing each technology against Zeno™ Walkway that serves as the reference system. The results are presented separately for both Single-Task and Dual-Task walking trials. Also, Table 4 presents the capability of these various sensing technologies in capturing trends in gait markers relative to those obtained from the Zeno™ Walkway via correlation analysis for both Single-Task and Dual-Task trials.

**Figure 5:**
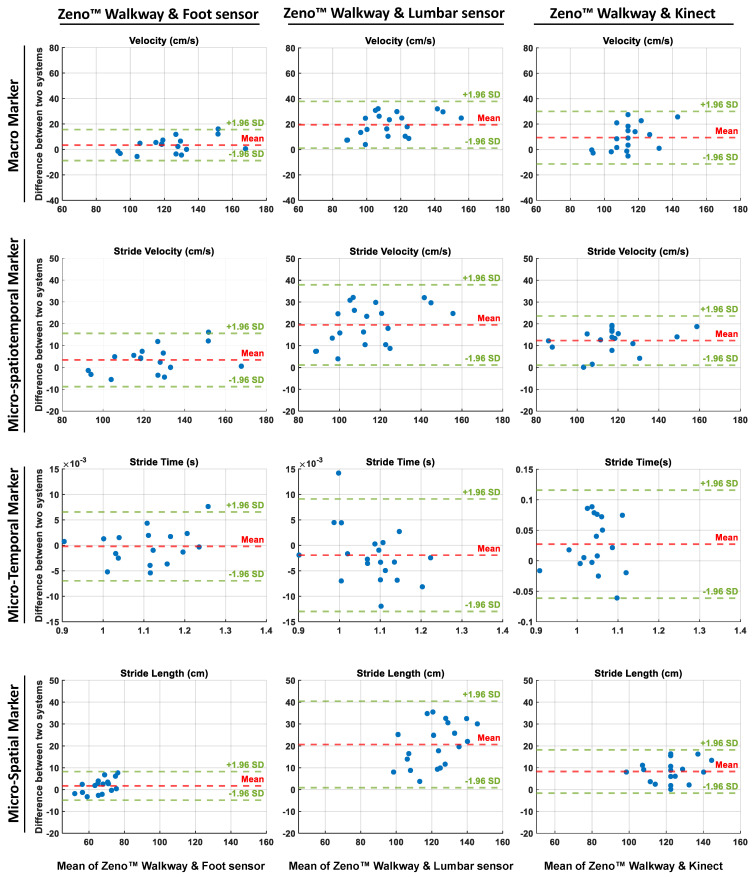
Bland–Altman plots comparing gait markers derived from the Zeno™ Walkway with those obtained from foot-mounted sensors (left column), lumbar-mounted sensors (middle column), and Azure Kinect (right column). Each row represents a specific category of gait markers: macro (velocity), micro-spatiotemporal (stride velocity), temporal (stride time), and spatial (stride length). The x-axes show the mean of the two measurement systems, while the y-axes indicate their differences. Red dashed lines represent the mean bias, and green dashed lines indicate the 95% limits of agreement (±1.96 SD). These plots illustrate the level of agreement and systematic bias across different sensor modalities for representative gait markers.

Moreover, to assess the agreement between the gait markers extracted from the Zeno™ Walkway and those derived from alternative sensor technologies, Bland–Altman analyses were conducted. These analyses evaluated the mean differences and LoA between the Zeno™ Walkway and foot-mounted sensors, lumbar-mounted sensors, and Azure Kinect, across macro and micro-temporal, micro-spatial, and micro-spatiotemporal gait marker categories. Figure 5 illustrates the Bland–Altman plots, where the first, second, and third columns correspond to the agreement between Zeno™ Walkway and foot-mounted sensors, lumbar-mounted sensors, and Azure Kinect, respectively. For each sensor comparison, the plots display the Bland–Altman analysis of gait markers selected as the best representatives in their respective categories: velocity for macro markers, stride velocity for micro-spatiotemporal markers, stride time for temporal markers, and stride length for spatial markers.

### 3.1. Overall Agreement Between Sensing Technologies and Zeno™ Walkway

In the Single-Task condition, the MAE for gait markers derived from foot-mounted IMU sensors ranged from 0.00±0.00 to 6.12±4.29 (mean±SD), with the lowest errors observed in temporal parameters such as step time and stride time and the highest error found in stride length. For the lumbar-mounted sensors, MAEs ranged from 0.00±0.00 for step time to 28.84±17.46 for average velocity, indicating markedly lower accuracy compared to foot-mounted sensors. The Azure Kinect demonstrated MAEs between 0.01±0.01 and 6.07±4.01, with the lowest errors for step and stride times and the highest for stride velocity. Under the Dual-Task condition, similar trends were observed, although errors were generally slightly higher. This decrease in accuracy may be partly explained by slower walking speeds during dual-tasking, which reduce the number of gait cycles captured within a given time window. At lower speeds, maintaining the same level of precision would likely require a higher sampling frequency to preserve the temporal resolution observed in the Single-Task condition. Foot-mounted sensors exhibited MAEs from 0.00±0.00 for step time to 8.13±5.75 for stride length, lumbar-mounted sensors ranged from 0.00±0.00 to 19.91±11.75 (again highest in average velocity), and Azure Kinect ranged from 0.01±0.01 for stride time to 6.91±5.28 for stride length.

Correlation analyses revealed moderate-to-strong agreement between the three sensor modalities and Zeno™ Walkway, with the highest correlations observed for foot-mounted sensors. In Single-Task trials, foot-mounted sensors achieved correlations ranging from r=0.92 (p<0.001) for step and stride length to r=1 (p<0.001) for step and stride time and cadence. Lumbar-mounted sensors exhibited perfect correlations for step and stride time (r=1, p<0.001) but substantially weaker correlations for other temporal markers, including swing and single support time (r=0.20, p=0.400). Azure Kinect correlations ranged from r=0.68 (p=0.025) for single support time to r=0.98 (p<0.001) for stride velocity, with all markers significantly correlated. In Dual-Task trials, foot-mounted sensors continued to demonstrate strong correlations across all markers, while lumbar-mounted sensors yielded significant correlations for 8 out of 11 markers, ranging from r=0.52 (p=0.020) for stance time to r=1 (p<0.001) for step and stride time; however, swing, single support, and double support times remained weak (r=0.12, p=0.630 for swing and single support, and r=0.34, p=0.150 for double support). Azure Kinect correlations in the Dual-Task condition ranged from r=0.70 (p=0.001) for swing and single support times to r=0.98 (p<0.001) for stride velocity, with all correlations statistically significant. Overall, foot-mounted IMU sensors and the Azure Kinect consistently demonstrated high agreement with Zeno™ Walkway across both conditions, while the lumbar-mounted sensor performed well for select markers (step and stride time) but showed reduced reliability for more detailed temporal and phase-specific gait parameters.

### 3.2. Macro-Level Gait Marker Agreement Across Technologies

The comparison of macro-level gait markers, specifically average velocity and cadence, between wearable sensors and the Azure Kinect relative to Zeno™ Walkway revealed clear performance differences across sensing modalities. In the Single-Task condition, the mean±SD of average velocity and cadence measured by Zeno™ Walkway were 125.09±21.14cm/s and 111.85±8.50steps/min, respectively. Corresponding values were 124.43±23.30 and 112.32±11.65 for foot-mounted wearable sensors, 95.23±13.83 and 125.53±12.37 for the lumbar-mounted sensor, and 127.18±25.18 and 119.67±9.10 for the Azure Kinect. Analysis of the MAE for average velocity demonstrated that foot-mounted sensors (0.68±1.76) and Azure Kinect (2.50±1.56) closely approximated the Zeno™ Walkway reference, whereas the lumbar-mounted sensor exhibited a markedly higher MAE of 28.84±17.46, reflecting poor agreement.

Dual-Task trials showed similar patterns but with expected reductions in overall gait performance; Zeno™ Walkway-measured average velocity decreased to 108.68 ± 20.43 cm/s, with corresponding values of 105.97±21.51cm/s (MAE =3.17±2.68) for foot-mounted sensors, 87.77±15.42cm/s (MAE =19.91±11.75) for lumbar-mounted sensors, and 101.87±17.05cm/s (MAE =5.87±3.82) for Azure Kinect. Correlation analyses reinforced these findings: in Single-Task trials, foot-mounted sensors exhibited higher correlation with Zeno™ Walkway for average velocity and cadence (r=0.0.99, p<0.001 and r=1, p<0.001) than Azure Kinect (r=0.85, p<0.001 and r=0.94, p<0.001) and lumbar-mounted sensors (r=0.93, p<0.001 and r=0.92, p<0.001). In the Dual-Task condition, foot-mounted sensors achieved the strongest correlations for both markers (r=0.99 and r=1, p<0.001), followed by Azure Kinect (r=0.97 and r=0.95, p<0.001) and lumbar-mounted sensors (r=0.95 and r=0.93, p<0.001).

As Figure 5 shows, Bland–Altman analysis further supported these results: foot-mounted sensors displayed a bias profile similar to Azure Kinect, with minimal mean differences, narrow limits of agreement, and randomly distributed differences, indicating consistent agreement with Zeno™ Walkway. In contrast, the lumbar-mounted sensor showed substantial mean bias and broader limits of agreement, highlighting systematic underestimation and reduced reliability in measuring macro-level gait velocity.

### 3.3. Micro-Temporal Gait Marker Agreement Across Technologies

For gait cycle duration (stride time), the mean ± SD was 1.08 ± 0.08 s (MAE = 0.00 ± 0.00) for the Zeno™ Walkway, 1.08±0.08 (MAE =0.00±0.00) for the foot-mounted wearable sensors, 1.08±0.00 (MAE = 0.00 ± 0.00) for the lumbar-mounted sensor, and 1.06±0.07 (MAE =0.01±0.01) for the Azure Kinect. For subphases of the gait cycle, including step time, stance time, and swing time, both the foot-mounted sensors and Azure Kinect demonstrated very low errors—often 0.00±0.00 or near zero—indicating high accuracy in the Single-Task condition. In contrast, the lumbar-mounted sensor showed higher MAEs for most of these markers, with the exception of step time, which remained accurate at 0.00±0.00. A similar trend was observed in the Dual-Task condition, where foot-mounted sensors and Azure Kinect maintained low MAEs for stride time, step time, stance, and swing times, while lumbar-mounted sensors exhibited consistently higher errors for all except step time. The high standard deviation in lumbar stance time reflects consistent underestimation relative to Zeno™ Walkway values rather than isolated outliers. Upon further investigation, it is revealed that this variability arises from limitations in estimating gait subphases using lumbar IMUs, which rely on indirect approximations. In contrast, foot-mounted IMUs offer more precise detection of contact events, leading to lower variance and higher agreement. For additional micro-temporal markers, such as single and double support times, foot-mounted sensors again achieved the lowest MAEs (0.01±0.01 and 0.03±0.02, respectively), followed by Azure Kinect (0.04±0.02 and 0.06±0.03), whereas the lumbar-mounted sensor showed markedly higher errors (0.37±0.21 and 0.73±0.31) in the Single-Task condition; these differences persisted in the Dual-Task condition.

Correlation analyses further confirmed these patterns: for stride time, both foot-mounted and lumbar-mounted sensors achieved perfect correlation with Zeno™ Walkway (r=1, p<0.001), while Azure Kinect showed slightly lower but still strong correlation (r=0.88, p<0.001) in Single-Task trials. For step time, correlations were similarly perfect for foot- and lumbar-mounted sensors (r=1, p<0.001) and slightly lower for Azure Kinect (r=0.92, p<0.001). For other temporal markers—stance, swing, single support, and double support times—foot-mounted sensors showed the highest correlations (r=0.99, 0.96, 0.96, and 0.98, p<0.001 for all), followed by Azure Kinect (r=0.80, p=0.007; r=0.91, p<0.001; r=0.68, p=0.025; and r=0.82, p=0.003, respectively). The lumbar-mounted sensor showed substantially weaker performance, with correlations of r=0.58 (p=0.010) for stance time, r=0.20 (p=0.400) for both swing and single support times, and r=0.54 (p=0.020) for double support time. In the Dual-Task condition, stride and step time correlations remained perfect (r=1, p<0.001) for foot-mounted and lumbar sensors and slightly lower for Azure Kinect (r=0.90, p<0.001 for stride time; r=0.94, p<0.001 for step time), while other temporal markers continued to show superior correlations for foot-mounted and Kinect sensors compared to lumbar-mounted sensors.

Bland–Altman analysis supported these findings: stride time exhibited strong agreement across all technologies, with foot-mounted and lumbar-mounted sensors displaying minimal dispersion (on the order of $10^{-3}$ s), while Azure Kinect showed slightly wider variability (on the order of $10^{-1}$ s), indicating a small but notable bias compared to the reference system.

### 3.4. Micro-Spatial Gait Marker Agreement Across Technologies

For spatial gait markers, specifically step and stride length, all three sensing technologies exhibited higher MAEs compared to their corresponding temporal markers (step and stride time). In the Single-Task condition, the mean±SD of step length and stride length were 65.49±6.42 cm (MAE =3.07±2.06) and 130.98±12.84 cm (MAE =6.12±4.29) for foot-mounted wearable sensors, 55.68±5.89 cm (MAE =10.42±4.86) and 111.82±12.00 cm (MAE =20.67±10.09) for lumbar-mounted wearable sensors, and 61.38±8.89 cm (MAE =4.83±3.28) and 120.98±19.77 cm (MAE =5.85±3.85) for Azure Kinect. In comparison, Zeno™ Walkway recorded 66.62±10.46 cm for step length and 133.55±14.67 cm for stride length. In the Dual-Task condition, similar patterns were observed, with spatial errors remaining larger than temporal errors. Specifically, step and stride lengths were 60.39±7.93 cm (MAE =3.99±2.77) and 124.32±17.38 cm (MAE =8.13±5.75) for foot-mounted sensors, 52.48±5.13 cm (MAE =8.64±4.46) and 105.55±10.44 cm (MAE =16.80±9.18) for lumbar-mounted sensors, and 60.68±10.63 cm (MAE =5.12±3.42) and 113.79±14.57 cm (MAE =6.91±5.28) for Azure Kinect, while Zeno™ Walkway measured 62.13±8.66 cm and 124.32±17.38 cm for step and stride length, respectively.

Correlation analyses showed that, in Single-Task trials, step and stride lengths measured via foot-mounted sensors and Azure Kinect were strongly correlated with Zeno™ Walkway (r=0.92, p<0.001 for both step and stride length with foot-mounted sensors; r=0.92, p<0.001 and r=0.90, p<0.001 with Azure Kinect). Lumbar-mounted sensors showed lower correlations (r=0.84, p<0.001 for step length and r=0.83, p<0.001 for stride length). In the Dual-Task condition, Azure Kinect demonstrated the highest correlations for both step and stride length (r=0.92 and r=0.96, p<0.001), followed by foot-mounted sensors (r=0.85 and r=0.84, p<0.001) and lumbar-mounted sensors (r=0.89 and r=0.88, p<0.001).

Bland–Altman analysis further confirmed these findings: stride length measured via lumbar-mounted sensors showed wider limits of agreement and evident systematic bias compared to Zeno™ Walkway, while foot-mounted sensors and Azure Kinect demonstrated narrower limits of agreement and minimal bias, with data points scattered randomly around the mean difference line (Figure 5).

### 3.5. Micro-Spatiotemporal Gait Marker Agreement Across Technologies

For the spatiotemporal marker of stride velocity, all the other sensor technologies underestimated the values than the Zeno™ Walkway system. The stride velocity reported 125.15±21.14 cm/s via Zeno™ Walkway while this marker was 122.56±18.86 cm/s (5.50 ± 4.31), 104.29±16.49 cm/s (19.49±9.39), and 120.84±25.92 cm/s (6.07±4.01) via wearable sensors mounted on the feet and lumbar, and Azure Kinect, respectively, in Single-Task condition. Similarly, these three technologies reported lower stride velocity than Zeno™ Walkway with 107.17±20.98 cm/s (6.66±4.72) via feet-mounted wearable sensors, 91.50±14.49 cm/s (14.92±8.50) via lumbar-mounted wearable sensors, and 105.68±14.79 cm/s (6.98±1.75) via Azure Kinect camera, while it was measured 108.85±20.43 cm/s by the Zeno™ Walkway reference system in the Dual-Task condition too. These results showed that the stride velocity measured by the feet-mounted wearable sensor and the Azure Kinect camera resulted in high accuracy in comparison to the Zeno™ Walkway, while the lumbar-mounted wearable sensor could not be measured as accurately as these two technologies.

Regarding the correlation metric, they also confirmed the higher capability of feet-mounted wearable sensor and Azure Kinect camera for measuring the stride velocity with r=0.96 (p<0.001) and r=0.98 (p<0.001) in the Single-Task condition while it was r=0.92 (p<0.001) when measured via lumbar-mounted wearable sensors in the same condition. Similarly, in the Dual-Task condition, they were r=0.93 (p<0.001), r=0.98 (p<0.001), and r=0.93 (p<0.001) for feet-mounted wearable sensors, Azure Kinect camera, and lumbar-mounted wearable sensors, respectively.

The Bland–Altman analysis also resulted in the lowest bias and narrowest limit of agreement for the feet-mounted wearable sensors, followed by results of Azure Kinect, as presented in Figure 5. In comparison, the measured stride velocity showed higher bias and wider agreement lines, which showed the lower capability of lumbar-mounted wearable sensors than foot-mounted wearable sensors and the Azure Kinect camera for measuring the spatiotemporal marker of stride velocity in comparison to the Zeno™ Walkway as the reference system.

### 3.6. Correlation vs. Mean Absolute Error Percentage Analysis

**Figure 6:**
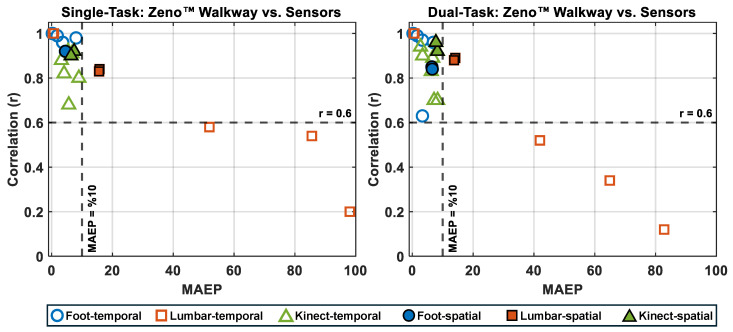
Correlation (*r*) versus Mean Absolute Error Percentage (MAEP) for temporal and spatial micro gait markers measured by foot-mounted, lumbar-mounted, and Kinect sensors compared to the Zeno™ Walkway reference. Unfilled symbols represent temporal markers and filled symbols represent spatial markers. The left panel shows Single-Task walking results, and the right panel shows Dual-Task walking results. Markers positioned toward the top-left quadrant (high *r*, low MAEP) indicate better agreement with Zeno™ Walkway. Horizontal and vertical dashed lines represent thresholds of r=0.6 and MAEP = 10%, respectively.

To further evaluate micro gait markers, the relationship between correlation and mean absolute error percentage (MAEP) was visualized for each technology relative to Zeno™ Walkway (Figure 6). In both Single-Task and Dual-Task conditions, foot-mounted wearable sensors consistently demonstrated lower MAEP (below 10%) and higher correlation coefficients (greater than r=0.6) across all micro-temporal and micro-spatial gait markers. Azure Kinect exhibited comparable performance, with similarly low MAEP and high correlation, albeit slightly lower than foot-mounted sensors for some markers. In contrast, lumbar-mounted sensors displayed reduced performance, with several markers—particularly stance time, swing time, single support time, and double support time—showing MAEP values exceeding 10% and correlation coefficients below r=0.6. This performance gap remained evident in Dual-Task conditions, highlighting the superior reliability of foot-mounted sensors and Azure Kinect for capturing fine-grained gait characteristics across varying cognitive loads.

## 4. Discussion

Gait is increasingly recognized as valuable clinical markers across a broad range of applications, from fall risk assessment and rehabilitation monitoring to early detection of neurodegenerative diseases such as Alzheimer’s and Parkinson’s disease [2,28,48]. Advances in sensing technologies—particularly wearable IMUs and markerless depth cameras—offer promising alternatives to traditional electronic walkways like Zeno™ Walkway, which remain the clinical gold standard for gait analysis. These emerging tools have the potential to enable scalable, cost-effective, and remote assessments, particularly in real-world settings where controlled laboratory conditions are impractical. This study directly compared three sensing technologies—APDM wearable sensors (foot- and lumbar-mounted) and the Azure Kinect depth camera—against Zeno™ Walkway across Single- and Dual-Task walking conditions in a real-world clinical environment. A key methodological strength was the development of a custom hardware system enabling precise temporal synchronization across all devices. We analyzed 11 gait markers, including macro-level metrics such as average velocity and cadence and micro-level temporal, spatial, and spatiotemporal parameters, to assess each technology’s agreement with Zeno™ Walkway using correlation, mean absolute error, and Bland–Altman analyses.

### 4.1. Main Findings

The findings consistently showed that foot-mounted wearable sensors and the Azure Kinect outperformed lumbar-mounted sensors across both Single- and Dual-Task conditions. Foot-mounted sensors achieved near-perfect agreement with Zeno™ Walkway for macro and temporal markers and high accuracy for spatial and spatiotemporal markers. Azure Kinect performed comparably, showing strong correlations and low error for most markers despite operating in a complex clinical environment with multiple people in view. In contrast, lumbar-mounted sensors exhibited greater variability and underperformed, particularly for phase-specific temporal markers like swing time and support phases. These trends were further confirmed by visualization of correlation versus mean absolute error percentage, where foot-mounted sensors and Azure Kinect clustered in the high-correlation, low-error quadrant, while lumbar-mounted sensors frequently exceeded the 10% error threshold and fell below the acceptable correlation threshold (Figure 6).

### 4.2. Comparison with Previous Studies

Our study extends prior research on gait analysis by directly comparing three distinct sensing technologies—APDM wearable sensors mounted on the feet and lumbar, the Azure Kinect depth camera, and the Zeno™ Walkway electronic walkway—within the same experimental framework. Previous studies have typically examined only two technologies at a time, such as depth cameras versus infrared motion capture systems [24,25,26], depth cameras versus electronic walkways like GAITRite [22,23,27], or wearable sensors versus GAITRite [15,16,17,18,19,20,21] (see Table 5). To our knowledge, no prior work has concurrently evaluated all three modalities against a clinical gold standard, allowing our findings to provide a more comprehensive perspective on the strengths and limitations of each technology.

A major methodological advancement of this study is the real-world clinical setting used for data collection. Unlike earlier studies that ensured a controlled environment with only one individual visible to the depth camera [22,23,24,25,26,27], our recordings included additional personnel such as caregivers and staff, reflecting typical clinical conditions. This introduces realistic challenges for markerless tracking but enhances ecological validity and supports future deployment in routine clinical or home settings. Additionally, we developed custom hardware to achieve precise temporal synchronization across all devices, an improvement over earlier studies that relied on manual alignment or approximate step-count matching [17,20].

In terms of test conditions, most prior studies conducted evaluations exclusively under Single-Task walking scenarios, typically assessing participants at their preferred, self-selected walking speed. Some investigations extended this by incorporating variations within the Single-Task paradigm, such as treadmill-based assessments [26] or trials requiring participants to walk at slow, normal, and fast speeds [18,27]. In contrast, only one previous study [22], alongside our work, evaluated both Single-Task and Dual-Task conditions, with the latter incorporating a cognitive challenge—normal-speed walking combined with backward counting. Including a Dual-Task condition allows for assessment of each system’s robustness under increased cognitive load, which is critical for understanding their applicability in real-world clinical and home settings where multitasking is common [37].

Another distinguishing feature is the breadth of gait markers evaluated: we analyzed 11 markers spanning macro (e.g., cadence, average velocity) and micro domains (temporal, spatial, and spatiotemporal markers), whereas previous studies generally focused on a limited set of two to nine markers [18]. This comprehensive analysis enables deeper insights into marker-specific performance, particularly for markers like stride velocity that have rarely been studied [28].

When comparing our results with previous findings, we observed several consistencies as well as important differences. For macro-level markers, such as average velocity and cadence, our foot-mounted sensors and Azure Kinect produced mean absolute errors and correlations similar to or better than those previously reported. For example, Lanotte et al. [18] reported an MAE of 11.55 cm/s for average velocity using combined foot and lumbar sensors, while our foot-mounted configuration achieved lower errors, highlighting the importance of sensor placement. Similarly, prior evaluations of Azure Kinect in controlled settings demonstrated high correlations for average velocity and cadence [23]; our results confirmed comparable accuracy in a less controlled clinical environment, underscoring its robustness under real-world conditions.

For micro-temporal markers, previous studies using APDM sensors reported high accuracy for stride and step time, with broader variability in stance, swing, and support phases [18,20]. Our results align with these patterns but further demonstrate that foot-mounted sensors maintain high accuracy across all temporal phases, while lumbar-mounted sensors show notable reductions in agreement, particularly for swing and support times. Importantly, no prior studies have benchmarked Azure Kinect for micro-temporal markers against an electronic walkway, making our findings a novel contribution that expands understanding of this technology’s capabilities. Spatial markers, including step and stride length, exhibited similar trends. Prior work reported correlations of 0.93–0.98 for foot-mounted sensors and lower values around 0.68–0.70 for lumbar-mounted sensors [18,20]. Our results confirmed this performance gradient and showed that Azure Kinect achieved strong correlations (≈0.92) despite being evaluated with a single camera in an unconstrained clinical environment, compared to prior studies using multiple cameras in laboratory settings [23]. These differences suggest that Azure Kinect remains viable for clinical applications even under more realistic constraints, though additional work may optimize tracking algorithms for multi-person scenes.

Collectively, these comparisons highlight both the strengths of our approach and the limitations of earlier studies. By incorporating multiple technologies, broader marker coverage, and realistic data collection conditions, we provide a more nuanced assessment of each sensor’s clinical applicability. Our findings reinforce the suitability of foot-mounted sensors and Azure Kinect for accurate and scalable gait analysis, while also demonstrating the limitations of lumbar-mounted sensors for fine-grained markers. This evidence supports their use in future clinical trials and informs decisions about technology adoption in routine gait assessments, especially for early detection and monitoring of neurodegenerative disorders.

### 4.3. Limitations and Future Directions

Although the sample size in this study was modest, it is comparable to that used in many prior validation studies of gait technologies (e.g., [22,24,25]) and was sufficient to demonstrate clear performance trends across the evaluated systems. Importantly, our cohort included both individuals with mild cognitive impairment and cognitively healthy controls, enabling us to capture variability across different cognitive states. While longer walking distances offer more comprehensive gait analysis, our study used a 7 m forward path (15 m total including return) due to clinical space limitations. This setup aligns with common clinical protocols, where 6 and 10 m walk tests are standard and have shown reliable accuracy for gait assessment [49,50,51]. Thus, our design reflects real-world clinical applicability. Also, while Azure Kinect demonstrated strong performance, its practical limitations—such as fixed camera height and angle, sensitivity to lighting, and a need for >6 m of unobstructed space—may restrict its use in some home or clinic settings. These factors can hinder generalizability without careful setup. In contrast, wearable IMUs are more portable and environment-flexible, making them suitable alternatives or complements in diverse real-world applications. Another area for growth is the range of gait tasks examined. This study focused on straight-line walking, which is widely used and directly comparable to prior work, but additional tasks such as curved walking, Timed Up and Go, or dual-task paradigms with higher cognitive demands could provide richer insights into functional mobility and better reflect real-world conditions. Building on these encouraging results, future work will expand to larger and more diverse populations and incorporate complex walking paths and functional mobility tasks.

## 5. Conclusions

This study compared three sensing technologies—APDM wearable sensors (feet and lumbar), Azure Kinect, and a computerized electronic walkway (Zeno™ Walkway)—for gait analysis in 18 older adults (mean age: 70.06 ± 9.45). Unlike prior work, this study simultaneously evaluated all three technologies under real-world clinical conditions where multiple individuals were present in the camera’s field of view, employed a custom-designed hardware synchronization system for precise temporal alignment, and analyzed an extensive set of detailed gait markers spanning macro, micro-temporal, micro-spatial, and micro-spatiotemporal domains. Results showed that APDM foot-mounted sensors offered the lowest MAE and highest correlation with Zeno™ Walkway (MAE = 0.00 ± 0.00, *r* = 1), while Azure Kinect demonstrated high promising performance with greater ease of use and lower cost (MAE = 0.01 ± 0.01, r = 0.98). These findings highlight the feasibility of both foot-mounted wearable sensors and Azure Kinect as scalable alternatives to electronic walkways for accurate gait assessment. Beyond their value in research, these technologies have broad clinical relevance, offering potential for fall risk assessment, rehabilitation monitoring, and early detection of various conditions, including, but not limited to, neurodegenerative diseases.

## Figures and Tables

**Table 1:** Participant demographics for all recruited participants and those included in the final analysis. Values are presented as numbers or Mean ± SD.

Variable	All Participants (n = 20)	Included in Analysis (n = 18)
Sex (Female/Male)	12/8	10/8
Age (years)	68.85±8.86	70.06±9.45
Height (m)	1.64±0.09	1.70±0.34
Weight (kg)	76.95±15.77	76.65±15.62
BMI (kg/m^2^)	29.07±7.47	28.65±7.20
Leg Length (m)	0.94±0.40	0.94±0.42
MoCA Score	26.05±2.91	26.17±2.90

***BMI*** = Body Mass Index; ***MoCA*** = Montreal Cognitive Assessment.

**Table 2:** Formulas used to calculate temporal and spatial gait markers.

	Gait Marker	Formula
**Temporal Gait Markers**		
Applied to all Technologies	Step Time	$HS_{R}\left(i\right)-HS_{L}\left(i\right)$
	Stride Time	$HS_{R}\left(i+1\right)-HS_{R}\left(i\right)$
	Stance Time	$TO_{R}\left(i\right)-HS_{R}\left(i\right)$
	Swing Time	StrideTime−StanceTime
	Single Support Time	$HS_{R}\left(i+1\right)-TO_{L}\left(i\right)$
	Initial Double Support Time (DST)	$TO_{L}\left(i\right)-HS_{R}\left(i\right)$
	Terminal Double Support Time (DST)	$TO_{R}\left(i\right)-HS_{L}\left(i+1\right)$
	Double Support Time	InitialDST+TerminalDST
**Spatial Gait Markers**		
Foot Sensors	Step Length	$SL=K\sqrt[{4}]{a_{\max}-a_{\min}}$, where $a_{\max}$ and $a_{\min}$ are the maximum and minimum vertical acceleration values during one step.
	Stride Length	$Stride=K\sqrt[{4}]{a_{\max}-a_{\min}}$, applied over one stride.
Lumbar Sensor	Step Length	$SL=2\sqrt{2lh-h^{2}}$ (inverted pendulum model), where *l* is foot length and *h* is vertical displacement during the step [42].
	Stride Length	$Stride=Step_{R}+Step_{L}$
Azure Kinect	Step Length	$\left|z\left(HS_{R}\left(i\right)\right)-z\left(HS_{L}\left(i\right)\right)\right|$
	Stride Length	$\left|z\left(HS_{R}\left(i+1\right)\right)-z\left(HS_{R}\left(i\right)\right)\right|$

***HS*** = Heel Strike; ***TO*** = Toe-Off; ***R*** = Right foot; ***L*** = Left foot; ***i*** = Current event; ***i + 1*** = Next event.

**Table 3:** Comparison of gait markers in *Single-Task* and *Dual-Task* conditions, including mean and mean absolute error (MAE) between Zeno™ Walkway and other sensors. Macro gait markers are separated from micro gait markers by a line.

Gait Marker	Zeno™	Foot	Zeno™—Foot	Lumbar	Zeno™—Lumbar	Kinect	Zeno™—Kinect
	**Mean ± SD**	**Mean ± SD**	**MAE (Mean ± SD)**	**Mean ± SD**	**MAE (Mean ± SD)**	**Mean ± SD**	**MAE (Mean ± SD)**
**Single-Task**							
Velocity (cm/s)	125.09±21.14	124.43±23.30	0.68±1.76	95.23±13.83	28.84±17.46	127.18±25.18	2.5±1.56
Cadence (steps/min)	111.85±8.50	112.32±11.65	1.52±1.57	125.53±12.37	10.37±7.23	119.67±9.10	5.77±3.98
Step time (s)	0.54±0.04	0.54±0.04	0.00±0.00	0.54±0.04	0.00±0.00	0.50±0.04	0.03±0.02
Stride time (s)	1.08±0.08	1.08±0.08	0.00±0.00	1.08±0.08	0.00±0.00	1.06±0.07	0.01±0.01
Stance time (s)	0.71±0.07	0.72±0.07	0.01±0.01	0.35±0.27	0.37±0.21	0.55±0.04	0.13±0.04
Swing time (s)	0.37±0.03	0.36±0.03	0.01±0.01	0.74±0.23	0.37±0.21	0.38±0.04	0.03±0.01
Single support time (s)	0.37±0.03	0.36±0.03	0.01±0.01	0.74±0.23	0.37±0.21	0.38±0.03	0.04±0.02
Double support time (s)	0.33±0.07	0.36±0.07	0.03±0.02	0.56±0.22	0.73±0.31	0.26±0.05	0.06±0.03
Step length (cm)	66.62±10.46	65.49±6.42	3.07±2.06	55.68±5.89	10.42±4.86	61.38±8.89	4.83±3.28
Stride length (cm)	133.55±14.67	130.98±12.84	6.12±4.29	111.82±12.00	20.67±10.09	120.98±19.77	5.85±3.85
Stride velocity (cm/s)	125.15±21.14	122.56±18.86	5.50±4.31	104.29±16.49	19.49±9.39	120.84±25.92	6.07±4.01
**Dual-Task**							
Velocity (cm/s)	108.68±20.43	105.97±21.51	3.17±2.68	87.77±15.42	19.91±11.75	101.87±17.05	5.87±3.82
Cadence (steps/min)	104±10.71	103.22±9.68	0.75±0.98	111.45±14.02	7.41±10.50	102.37±7.86	3.10±2.23
Step time (s)	0.58±0.06	0.58±0.06	0.00±0.00	0.59±0.06	0.00±0.00	0.57±0.04	0.02±0.01
Stride time (s)	1.16±0.12	1.16±0.12	0.00±0.01	1.17±0.12	0.01±0.01	1.12±0.14	0.01±0.01
Stance time (s)	0.78±0.09	0.79±0.09	0.01±0.01	0.47±0.29	0.33±0.23	0.72±0.13	0.09±0.05
Swing time (s)	0.39±0.05	0.38±0.05	0.01±0.01	0.70±0.25	0.32±0.23	0.41±0.07	0.04±0.03
Single support time (s)	0.39±0.05	0.38±0.05	0.01±0.01	0.70±0.25	0.32±0.23	0.36±0.09	0.09±0.04
Double support time (s)	0.38±0.07	0.41±0.08	0.03±0.02	0.52±0.23	0.65±0.46	0.36±0.05	0.05±0.03
Step length (cm)	62.13±8.66	60.39±7.93	3.99±2.77	52.48±5.13	8.64±4.46	60.68±10.63	5.12±3.42
Stride length (cm)	124.32±17.38	120.78±15.85	8.13±5.75	105.55±10.44	16.80±9.18	113.79±14.57	6.91±5.28
Stride velocity (cm/s)	108.85±20.43	107.17±20.98	6.66±4.72	91.50±14.49	14.92±8.50	105.68±14.79	6.98±1.65

**Table 4:** Pearson Correlation (*r*) and significance (*p*-value) between Zeno™ Walkway and other sensor-based measurements across gait markers in *Single-Task* and *Dual-Task* conditions.

	Single-Task			Dual-Task		
**Gait Marker**	**Zeno™—Foot**	**Zeno™—Lumbar**	**Zeno™—Kinect**	**Zeno™—Foot**	**Zeno™—Lumbar**	**Zeno™—Kinect**
	**r (** ***p*** **-Value)**	**r (** ***p*** **-Value)**	**r (** ***p*** **-Value)**	**r (** ***p*** **-Value)**	**r (** ***p*** **-Value)**	**r (** ***p*** **-Value)**
Velocity (cm/s)	0.99 (*p* < 0.001)	0.93 (*p* < 0.001)	0.85 (*p* < 0.001)	0.99 (*p* < 0.001)	0.95 (*p* < 0.001)	0.97 (*p* < 0.001)
Cadence (steps/min)	1 (*p* < 0.001)	0.92 (*p* < 0.001)	0.94 (*p* < 0.001)	1 (*p* < 0.001)	0.93 (*p* < 0.001)	0.95 (*p* < 0.001)
Step time (s)	1 (*p* < 0.001)	1 (*p* < 0.001)	0.92 (*p* < 0.001)	1 (*p* < 0.001)	1 (*p* < 0.001)	0.94 (*p* < 0.001)
Stride time (s)	1 (*p* < 0.001)	1 (*p* < 0.001)	0.88 (*p* < 0.001)	1 (*p* < 0.001)	1 (*p* < 0.001)	0.90 (*p* < 0.001)
Stance time (s)	0.99 (*p* < 0.001)	0.58 (*p* = 0.010)	0.80 (*p* = 0.007)	0.99 (*p* < 0.001)	0.52 (*p* = 0.020)	0.89 (*p* < 0.001)
Swing time (s)	0.96 (*p* < 0.001)	0.20 (*p* = 0.400)	0.91 (*p* < 0.001)	0.97 (*p* = 0.026)	0.12 (*p* = 0.630)	0.70 (*p* = 0.016)
Single support time (s)	0.96 (*p* < 0.001)	0.20 (*p* = 0.400)	0.68 (*p* = 0.025)	0.97 (*p* < 0.001)	0.12 (*p* = 0.630)	0.70 (*p* = 0.001)
Double support time (s)	0.98 (*p* < 0.001)	0.54 (*p* = 0.020)	0.82 (*p* = 0.003)	0.96 (*p* < 0.001)	0.34 (*p* = 0.150)	0.83 (*p* = 0.001)
Step length (cm)	0.92 (*p* < 0.001)	0.84 (*p* < 0.001)	0.92 (*p* < 0.001)	0.85 (*p* < 0.001)	0.89 (*p* < 0.001)	0.92 (*p* < 0.001)
Stride length (cm)	0.92 (*p* < 0.001)	0.83 (*p* < 0.001)	0.90 (*p* < 0.001)	0.84 (*p* < 0.001)	0.88 (*p* < 0.001)	0.96 (*p* < 0.001)
Stride velocity (cm/s)	0.96 (*p* < 0.001)	0.92 (*p* < 0.001)	0.98 (*p* < 0.001)	0.93 (*p* < 0.001)	0.93 (*p* < 0.001)	0.98 (*p* < 0.001)

**Table 5:** Comparison of our study with previous studies.

Study	# Subjects	Sensor Technology	Gold Standard	Task Condition	Gait Markers r(n)
Clark et al., 2013 [24]	21	Kinect	Vicon	Single-Task	Step time, Step length, Gait speed, Stride time, Stride length, Foot swing velocity (6)
Dolatabadi et al., 2016 [22]	20	Kinect v2	GAITRite	Single-Task Dual-Task	Stance time, Step time, Step length, Velocity (4)
Moore et al., 2017 [15]	25	AX3 IMU	GAITRite	Single-Task	Step velocity, Step length, Step time, Swing time, Stance time (5)
Psaltos et al., 2019 [21]	40	Pressure insoles iPhone 8 Plus	GAITRite	Single-Task	Stance time, Swing time, Step time, Double support, Step length, Stride velocity, Stride time, Stride length (8)
Buckley et al., 2019 [16]	25	AX3 IMU	GAITRite	Single-Task	Step length, Step time, Cadence, Gait speed (4)
Steinert et al., 2019 [27]	44	Kinect v2 LG Nexus 5	GAITRite	Single-Task	Step time, Swing time, Stance time, Step length (4)
Muthukrishnan et al., 2020 [17]	15	APDM IMU	GAITRite	Single-Task	Step length, Step time (2)
Albert et al., 2020 [26]	5	Kinect v2 Azure Kinect	Vicon	Single-Task	Step length, Step time, Step width, Stride time (4)
Jacobs et al., 2021 [19]	25	FeetMe^®^ insoles	GAITRite	Single-Task	Stride length, Stride velocity, Stance time, Swing time, Cadence (5)
Rudisch et al., 2021 [20]	12	Physilog^®^5 APDM IMU	GAITRite	Single-Task	Stance time, Swing time, Step time, Step length, Stride velocity, Stride time, Double support, Stride length (8)
Guess et al., 2022 [25]	20	Azure Kinect	Vicon	Single-Task	Stride length, Stride time, Step width, Step length (4)
Lanotte et al., 2023 [18]	26	APDM IMU	GAITRite	Single-Task	Gait cycle, Cadence, Double support, Step time, Gait speed, Stride length, Stance time, Swing time, Single support (9)
Arizpe-Gómez et al., 2023 [23]	24	Azure Kinect	GAITRite	Single-Task	Step length, Cadence, Velocity (3)
**Our Study**	20	APDM IMU Azure Kinect	Zeno™ Walkway	Single-Task Dual-Task	Velocity, Cadence, Step time, Stride time, Stance time, Swing time, Single support time, Double support time, Step length, Stride length, Stride velocity (11)

## Data Availability

The original contributions presented in this study are included in the article. Further inquiries can be directed to the corresponding author.
